# Psychosocial factors associated with medication burden among community-dwelling older people with multimorbidity

**DOI:** 10.1186/s12877-023-04444-6

**Published:** 2023-11-14

**Authors:** Chen Yang, Song Zhu, Zhaozhao Hui, Yihan Mo

**Affiliations:** 1 School of Nursing, Sun Yat-sen University, Guangzhou, China; 2 Department of Thoracic Surgery, The First Affiliated Hospital, Sun Yat-sen University, Guangzhou, China; 3 School of Public Health, Xi’an Jiaotong University Health Science Centre, Xi’an, China; 4 Shaanxi Health Culture Research Center, Xianyang, China; 5 Cicely Saunders Institute of Palliative Care, Policy & Rehabilitation, King’s College London, London, UK

**Keywords:** Aged, Medication burden, Multimorbidity, Patient satisfaction, Self efficacy, Self-management

## Abstract

**Background:**

Older people with multimorbidity are often prescribed multiple medication treatments, leading to difficulties in self-managing their medications and negative experiences in medication use. The perceived burden arising from the process of undertaking medication self-management practices has been described as medication burden. Preliminary evidence has suggested that patients’ demographic and clinical characteristics may impact their medication burden. Little is known regarding how psychosocial factors affect medication burden in older people with multimorbidity. The aim of this study was to identify psychosocial factors associated with medication burden among community-dwelling older people with multimorbidity.

**Methods:**

This is a secondary analysis of a cross-sectional study. A total of 254 older people with three or more chronic conditions were included in the analysis. Participants were assessed for demographics, medication burden, psychosocial variables (depression, medication-related knowledge, beliefs, social support, self-efficacy, and satisfaction), disease burden, and polypharmacy. Medication burden was measured using items from the Treatment Burden Questionnaire. Univariate and multivariate linear regression models explored factors associated with medication burden.

**Results:**

The mean age of participants was 70.90 years. Participants had an average of 4.40 chronic conditions, and over one-third had polypharmacy. Multivariate analysis showed that the participants’ satisfaction with medication treatments (β = -0.32, p < 0.001), disease burden (β = 0.25, p = 0.009), medication self-efficacy (β = -0.21, p < 0.001), polypharmacy (β = 0.15, p = 0.016), and depression (β = 0.14, p = 0.016) were independently associated with medication burden. Other factors, including demographic characteristics, medication knowledge, medication beliefs, medication social support, and the number or specific types of chronic conditions, were not independently associated with medication burden.

**Conclusions:**

Poor medication treatment satisfaction, great disease burden, low medication self-efficacy, polypharmacy, and depression may increase individuals’ medication burden. Understanding psychosocial aspects associated with medication burden provides an important perspective for identifying older people who are overburdened by their medication treatments and offering individualised treatments to relieve their burden.

**Supplementary Information:**

The online version contains supplementary material available at 10.1186/s12877-023-04444-6.

## Background

Multimorbidity is highly prevalent in older people, with over half of individuals aged 65 years and older having two or more chronic conditions [1, 2]. Long-term management of chronic diseases usually needs the regular use of medications for controlling conditions, relieving symptoms, and preventing disease progression. A national population-based study in the United States found that over 80% of older people were prescribed at least one medication, and more than one-third was concurrently using ≥ 5 prescription medications [3].

Multimorbidity contributes to the use of multiple medications, which are linked to potentially inappropriate medications, adverse drug events and nonadherence [4]. Previous studies have shown that older people with multimorbidity had a 2–4 times higher risk of developing polypharmacy compared with those without multimorbidity [5, 6]. A large-scale retrospective longitudinal study in Spain found that older people’s medication-related problems (including duplicate therapy, drug-drug interactions and potentially inappropriate medications) increased with the number of chronic conditions [7].

Implementing medical treatment regimens and managing chronic diseases require a considerable expenditure of time and effort for older people with multimorbidity. The perceived burden arising from the process of undertaking treatment and self-care practices has been described as treatment burden [8]. One major aspect of treatment burden is medication burden, which is the workload imposed on patients resulting from the use of medications and its’ impact on patients’ health and well-being [9]. Medication burden is a multidimensional concept comprising different aspects of experiences with medication use. Maidment et al. [10] have summarised medication burden into five categories: (1) ambiguity burden (related to reviewing/reconciling medications within medication management), (2) concealment burden (due to a lack of information-giving), (3) unfamiliarity burden (resulting from not consistently seeing the same practitioner), (4) fragmentation burden (arising from challenges in interacting with fragmented healthcare services), (5) exclusion burden (i.e., older people and their carers are not recognised for their expertise in medication management and are not engaged in their healthcare decision-making). Medication burden can lead to nonadherence, suboptimal therapeutic outcomes, poor social functioning, and decreased quality of life [9, 11–13].

Medication burden is affected by the workload resulting from using medications and the personal capacity to manage the burden. According to the Cumulative Complexity Model, the successful self-management of chronic disease requires an adaptive balance between burden and capacity [14]. A patient’s internal and external resources, such as functional capacity, psychosocial functioning, and healthcare system support, determine one’s ability to manage medication burden and thus affect the extent to which the burden can have an impact on one’s health and well-being. As such, patients need adequate self-management capacity to cope with medication regimens and control medication burden. Medication self-management capacity refers to an individual’s “cognitive and functional ability to self-administer a medication regimen as it has been prescribed” [15]. It involves relevant knowledge and skills to successfully fill, understand, organise, take, monitor, and sustain medication use [16]. All demographic factors, such as patients’ age, sex, educational level, cultural background, and employment conditions, may affect their medication self-management capacity [17]. Personal attitudes and beliefs about medications also shape one’s readiness to cope with challenges and the burden of medication use. Furthermore, medication-related support from family, friends, or the healthcare system provides an opportunity to compensate for patients’ deficits in medication self-management, further enhancing their ability to manage medication treatments [18]. Therefore, considering medication self-management capacity is necessary to gain a comprehensive understanding of patients’ burdens associated with medication use.

Another important aspect closely linked to medication burden is satisfaction with medication treatment, which is the balance between patients’ expectations about the treatment, side effects, convenience of use, and perceived efficacy [19]. Patients tend to be unsatisfied with medication treatments because of perceived little health benefits from their treatments, medication side effects, or perceived inconvenience and difficulty of medication use [20]. Poor medication treatment satisfaction may lead to negative attitudes and beliefs towards taking medications, which are more likely to be viewed by patients as a source of burden [21]. Moreover, patients with poor medication treatment satisfaction are less likely to tolerate the burden associated with medication use and are unwilling to integrate medication treatments into their lives. Previous studies have established that poor satisfaction with medication treatments was associated with medication burden or overall treatment burden [19, 22].

Most existing research on medication burden is qualitative, centring on patients’ subjective experiences in medication use [23–25]. A few studies explored the associations between medication burden and demographic factors (such as age, sex, marital status, ethnic group, and employment) or clinical characteristics (including the number and duration of diseases) in patients with chronic conditions [12, 13, 26, 27]. Some studies were based on univariate analysis, which ignores the interdependence between different factors [13, 27]. The roles of psychosocial factors in affecting medication burden have not yet been explored. Studies have reported that healthcare professionals tend to ignore or overestimate patients’ capacity to self-manage and cope with treatment burden [28, 29]. A better understanding of psychosocial aspects associated with medication burden may provide an important perspective for minimising medication burden, promoting medication self-management capacity, and optimising adherence to medications. In this study, we specifically targeted older people with multimorbidity, a group of populations at high risk of being overburdened due to multiple medication treatments and their diminished capacity to self-manage these treatments. The overall aim of this study was to identify psychosocial factors that were associated with medication burden among older people with multimorbidity.

## Methods

### Study design

This is a secondary analysis using cross-sectional data that were collected between July and September 2019. The original study aimed to explore the predictors of medication adherence among community-dwelling older people with multimorbidity based on a social behaviour theory, i.e., the Information-Motivation-Behavioural Skills model [30, 31]. Participants’ medication adherence, treatment burden, disease burden, depression, and a range of medication-related variables were collected through face-to-face interviews. The study design has been described in detail elsewhere [30]. Written informed consent was obtained from each participant before starting the study. The cross-sectional data were anonymous, without personally identifiable information. The secondary analysis of the cross-sectional data was approved by the Survey and Behavioural Research Ethics Committee of the Chinese University of Hong Kong (Reference No. SBRE-21-0835).

### Participants and settings

A total of 254 participants were recruited using convenience sampling in two community health centres in Changsha, a city in the Northeastern Hunan Province of China. Eligibility criteria for the survey were subjects (1) aged 60 years or over, (2) having three or more chronic conditions from a list of 38 chronic diseases (for the list of diseases, please refer to the previously published study [30]), (3) prescribed one or more medication for chronic diseases, (4) able to self-administer medications, and (5) able to speak and understand Chinese. Exclusion criteria were having cognitive impairment (i.e., having a mini-cog score < 4 [32]), severe mental diseases, deafness, or participating in other research related to chronic disease management at the time of the study.

### Measurements

#### Demographic characteristics

Demographic information included age, sex, education level, marital status, monthly income, and insurance status.

#### Medication burden

Medication burden was measured using the first four items of the Treatment Burden Questionnaire (TBQ). This scale is a 15-item self-reported scale exploring a broad range of behavioural and emotional burden in the procedure of undertaking and engaging treatments [19, 33]. It measures an individual’s perceived burden related to taking medications, lifestyle changes, and the social and financial impacts of the treatments. The scale uses an 11-point Likert scale from 0 (not a problem) to 10 (big problem). A higher score indicates higher perceived burden. The Cronbach’s alpha for the scale in the current study was 0.886. The first four items of the TBQ specifically measure one’s medication burden, including the burden related to discomfort caused by medications, times to take medications on a daily basis, efforts to remember to take medications, and special precautions required when taking medications [33]. Participants’ medication burden was generated by summing up the scores of these four items, with a total score ranging from 0 to 40.

#### Medication knowledge

Medication knowledge was measured by the Patients’ Perceived Knowledge in Medication Use Questionnaire developed by Okere et al. [34]. It consists of five items related to individuals’ knowledge about medication use and drug interaction. It is scored with a 5-point Likert scale (1 = strongly disagree to 5 = strongly agree). Adding all item scores provides a total score ranging from 5 to 25. A higher score indicates a higher level of medication knowledge. The Cronbach’s alpha of this scale was 0.723 in the present study.

#### Medication beliefs

Medication beliefs were measured using the 18-item Beliefs about Medication Questionnaire (BMQ) [35]. The BMQ contains two sections with two subscales each: (1) BMQ-Specific, which evaluates one’s beliefs about the necessity of medication (5 items) and concerns about medications (5 items); and (2) BMQ-General assessing beliefs about the harm of medication (4 items) and overuse of medications by doctors (4 items). Each item is scored on a 5-point Likert scale (1 = strongly disagree to 5 = strongly agree). A total score indicates a stronger belief in the corresponding concepts in each subscale. The Cronbach’s alpha of the BMQ was 0.639 in the current study.

#### Medication social support

Medication social support was measured using the Medication Specific Social Support Questionnaire. Participants were asked how often others help with their medication taking [36]. This scale consists of eight items, each rated on a 5-point Likert scale from 0 (never) to 4 (very often). The scale score is the mean score obtained by dividing the total score by the number of items. A higher score indicates a higher level of medication social support. The Cronbach’s alpha of the scale in the present study was 0.878.

#### Medication self-efficacy

Medication self-efficacy was measured using a 13-item Self-Efficacy for Appropriate Medication Use Scale, which was developed to assess one’s confidence in taking medications under various challenging circumstances [37]. Participants were asked to rate each statement on a 3-point Likert scale ranging from 1 (not confident) to 3 (very confident). The total scores range from 13 to 39, with a higher score indicating a higher level of medication self-efficacy. Evidence has supported that the scale was a reliable and valid measure of self-efficacy in medication management [38]. The Cronbach’s alpha of the scale was 0.935 in the current study.

#### Medication treatment satisfaction

Medication treatment satisfaction was measured using the Treatment Satisfaction Questionnaire with Medication Version II [20]. It is an 11-item questionnaire comprising four dimensions (effectiveness, convenience, side effects, and global satisfaction). Each item is rated on a 5- or 7-point Likert scale. The score for each dimension is calculated by adding the scores of items contained in the dimension and then transforming the composite score into a value ranging from 0 to 100. In this study, we used the global satisfaction score to assess participants’ satisfaction with medication treatments. A higher score indicates higher satisfaction with medication use. The Cronbach’s alpha of the scale was 0.860 in the current study.

#### Depression

Depression was measured using the 9-item Patient Health Questionnaire [39]. This scale is designed to collect data regarding an individual’s depressive experiences in the last two weeks. The scale uses a 4-point Likert scale (0 = not at all to 3 = nearly every day). The total score ranges from 0 to 27, with a higher score indicating a higher level of depression. The Cronbach’s alpha of the scale was 0.759 in the present study.

#### Disease burden and polypharmacy

The Cumulative Illness Rating Scale-Geriatric was administered to assess an individual’s disease burden from 14 body systems [40]. This scale not only considers the presence of chronic conditions, but also scores the severity of each condition and the impacts of each condition on the individual. The severity of each body system is rated using a 5-point Likert scale (0 = no problem to 4 = extremely severe). The total score is generated by summing the severity scores of each system. A higher total score indicates higher disease burden (possible range, 0–56). Scoring of the scale was performed according to the guideline developed by Salvi et al. [41]. This scale has been validated among Chinese older adults [42].

Participants were also asked about the number of medications they were taking. Older people were regarded as having polypharmacy if they took five or more medications [43].

### Data analysis

Participants’ characteristics were summarised using means and standard deviations (SD) for continuous variables and frequencies and percentages for categorical variables. We compared medication burden across participants’ demographic and clinical characteristics using student’s t-test or one-way analysis of variance (ANOVA) for continuous data and Pearson’s 𝜒^2^ test for categorical data. Correlations between variables were examined using Pearson correlations. Univariate and multivariate linear regression models were employed to identify factors associated with medication burden. Multivariate linear regression models were adjusted for variables with p < 0.05 in the univariate regression analysis. To ensure the result consistency, we conducted an additional multivariate regression analysis that incorporated all psychosocial factors, variables that showed significance in the univariate analysis, as well as those were identified in previous studies as being associated with medication burden (i.e., age, sex, and marriage status) [44]. We also examined the association between medication burden and the presence of specific types of chronic conditions. Only chronic conditions with a prevalence of over 30% in our sample were included in the analysis to avoid overfitting with the regression models. The multicollinearity in the regression analysis was examined by the variance inflation factor (VIF), with VIF > 5 indicating collinearity [45]. Two variables with an asymmetrical distribution (i.e., disease burden and the number of conditions) were subject to logarithmic or reciprocal transformation prior to statistical inference. All statistical analyses were performed using IBM SPSS Statistics Version 26.0 (IBM Corp., Armonk, N.Y., USA). Statistical significance was set at a two-sided *p*-value < 0.05.

## Results

### Sample characteristics

A total of 254 participants were included in this analysis. The demographic and clinical characteristics are presented in Table 1. The mean age of participants was 70.90 (SD 6.82) years. There were slightly more female (57.9%; n = 147) than male participants (42.1%; n = 107). The majority of participants were married (74.8%). Nearly all participants were covered by medical insurance (98.8%). Participants had an average of 4.40 (SD 1.84) chronic conditions. The chronic conditions with a prevalence of over 30% were hypertension (75.2%), coronary heart disease (54.7%), chronic painful condition (36.2%), lipid disorder (33.9%), and glaucoma/cataract (31.5%). The mean number of prescribed medications was 3.78 (SD 2.48). Over one-third of participants (33.5%) had polypharmacy. Participants with chronic painful conditions, glaucoma/cataract, or polypharmacy had a statistically significantly higher level of medication burden compared to those without these conditions (all p-values < 0.05).

**Table 1:** Comparison of medication burden by participant characteristics (N = 254)

Characteristics	*n* (%)	Medication burden Mean (SD)	t/F/U	p
Age (years)				
60–74	178 (70.1)	7.21 (9.58)	0.31 ^a^	0.761
≥ 75	76 (29.9)	6.82 (8.94)		
Sex				
Male	107 (42.1)	6.54 (8.76)	-0.80 ^a^	0.428
Female	147 (57.9)	7.49 (9.81)		
Education level				
Elementary school or lower	94 (37.0)	7.90 (9.85)	0.60 ^b^	0.549
Junior or senior high school	115 (45.3)	6.48 (9.03)		
Technical school or college	45 (17.7)	6.96 (9.32)		
Marital status				
Married	190 (74.8)	6.76 (9.24)	-0.96 ^a^	0.339
Widow/Divorces	64 (25.2)	8.06 (9.79)		
Monthly income (CNY)				
< 1000	67 (26.4)	7.54 (9.87)	0.27 ^b^	0.765
1000–2999	84 (33.1)	7.37 (10.03)		
≥ 3000	103 (40.5)	6.57 (8.53)		
Hypertension				
No	63 (24.8)	8.95 (11.51)	1.57 ^a^	0.120
Yes	191 (75.2)	6.48 (8.51)		
Coronary heart disease				
No	115 (45.3)	5.49 (7.59)	7032.00 ^c^	0.087
Yes	139 (54.7)	8.42 (10.48)		
Chronic painful condition				
No	163 (64.2)	5.83 (8.13)	-2.67 ^a^	0.008^*^
Yes	91 (36.2)	9.34 (10.96)		
Lipid disorder				
No	168 (66.1)	7.47 (9.33)	0.90 ^a^	0.368
Yes	86 (33.9)	6.35 (9.49)		
Glaucoma/Cataract				
No	174 (68.5)	5.86 (8.35)	5827.00 ^c^	0.030 ^*^
Yes	80 (31.5)	9.76 (10.88)		
Polypharmacy				
No	169 (66.5)	4.46 (7.23)	3950.50 ^c^	< 0.001^***^
Yes	85 (33.5)	12.33 (10.89)		

*Note*: ^*^p < 0.05, ^***^p < 0.001

^a^ Independent t-test

^b^ One-way ANOVA

^c^ Mann-Whitney U test

### Correlations between medication burden and continuous variables

Table 2 presents the correlations between medication burden, psychosocial variables, disease burden, and the number of chronic conditions. The results showed that medication burden was statistically significantly negatively associated with medication knowledge, medication self-efficacy, and medication treatment satisfaction (r ranged from -0.15 to -0.52; all p-values < 0.05). Furthermore, medication burden was positively correlated with depression, disease burden, and the number of chronic conditions (r ranged from 0.39 to 0.46; all p-values < 0.001). There was no statistically significant correlation between medication burden and medication beliefs or medication social support (p > 0.05).

**Table 2:** Correlation between medication burden, psychosocial variables, disease burden and the number of chronic conditions

Variable	Mean (SD)	Correlation with medication burden	
		r	p
1. Medication knowledge	14.76 (3.99)	-0.15	0.014^*^
2. Medication motivation (necessity of medication)	17.41 (4.55)	0.00	0.985
3. Medication motivation (concerns about medication)	14.41 (5.26)	0.02	0.756
4. Medication motivation (harm of medication)	12.44 (2.69)	-0.01	0.827
5. Medication motivation (overuse of medication)	13.73 (3.19)	0.00	0.949
6. Medication social support	1.02 (0.90)	-0.11	0.073
7. Medication self-efficacy	29.87 (6.98)	-0.38	< 0.001^***^
8. Global satisfaction with medication treatments	68.73 (14.96)	-0.52	< 0.001^***^
9. Depression	4.22 (3.94)	0.39	< 0.001^***^
10. Disease burden	6.23 (2.53)	0.46	< 0.001^***^
11. Number of chronic conditions	4.40 (1.84)	0.41	< 0.001^***^
12. Medication burden	7.09 (9.38)	1	NA

*Note*: ^*^p < 0.05, ^***^p < 0.001

### Univariate and multivariate linear regression analyses for medication burden

On univariate analysis, coronary heart disease, chronic painful condition, glaucoma/cataract, polypharmacy, medication knowledge, medication self-efficacy, global satisfaction with medication treatments, depression, disease burden, and the number of chronic conditions were statistically significantly associated with medication burden (Table 3). These variables were therefore included in the multivariate linear regression model (Table 4). The multivariate analysis found that participants’ global satisfaction with medication treatments (β = -0.32, p < 0.001), disease burden (β = 0.25, p = 0.009), medication self-efficacy (β = -0.21, p < 0.001), polypharmacy (β = 0.15, p = 0.016), and depression (β = 0.14, p = 0.016) were independently associated with medication burden. The model explained a total variance of 45.6% [F (10, 243) = 20.346, p < 0.001]. No multicollinearity was detected, with VIF values < 5 for all variables. The additional regression model also showed that participants’ global satisfaction with medication treatments, disease burden, medication self-efficacy, polypharmacy, and depression were independently associated with medication burden (see Supplementary Table S1).

**Table 3:** Univariate linear regression examining factors associated with medication burden

Variables	Unstandardised coefficients		Standardised coefficients Beta	p
	B	Standard error		
Age	0.13	0.09	0.09	0.137
Sex (ref. male)				
Female	0.95	1.19	0.05	0.428
Education level (ref. elementary school or lower)				
Junior or senior high school	-1.43	1.31	-0.08	0.276
Technical school or college	-0.95	1.73	-0.04	0.578
Marital status (ref. married)				
Widow/Divorces	1.30	1.36	0.06	0.339
Monthly income (CNY; ref. <1000)				
1000–2999	-0.17	1.54	-0.01	0.913
≥ 3000	-0.96	1.48	-0.05	0.514
Hypertension (ref. no)				
Yes	-2.48	1.36	-0.11	0.069
Coronary heart disease (ref. no)				
Yes	2.93	1.17	0.16	0.013^**^
Chronic painful condition (ref. no)				
Yes	3.51	1.21	0.18	0.004^*^
Lipid disorder (ref. no)				
Yes	-1.12	1.24	-0.06	0.368
Glaucoma/Cataract (ref. no)				
Yes	3.90	1.25	0.19	0.002^**^
Polypharmacy (ref. no)				
Yes	7.87	1.15	0.40	< 0.001^***^
Medication knowledge	-0.36	0.15	-0.15	0.014^**^
Medication beliefs				
Necessity of medication	0.00	0.13	0.00	0.985
Concerns about medication	0.04	0.11	0.02	0.756
Harm of medication	-0.05	0.22	-0.01	0.827
Overuse of medication	-0.01	0.19	-0.00	0.949
Medication social support	-1.17	0.65	-0.11	0.073
Medication self-efficacy	-0.51	0.08	-0.38	< 0.001^***^
Global satisfaction with medication treatments	-0.33	0.03	-0.52	< 0.001^***^
Depression	3.50	0.52	0.39	< 0.001^***^
Disease burden	1.70	0.21	0.46	< 0.001^***^
Number of chronic conditions	9.69	1.35	0.41	< 0.001^***^

*Note*: ^*^p < 0.05, ^**^p < 0.01, ^***^p < 0.001

**Table 4:** Multiple linear regression examining factors associated with medication burden

Variables	Unstandardised coefficients		Standardised coefficients Beta	p
	B	Standard error		
Global satisfaction with medication treatments	-0.20	0.04	-0.32	< 0.001^***^
Disease burden	0.92	0.35	0.25	0.009^**^
Medication self-efficacy	-0.28	0.07	-0.21	< 0.001^***^
Polypharmacy (ref. no)	2.87	1.19	0.15	0.016^*^
Depression (ref. no)	1.22	0.50	0.14	0.016^*^
Coronary heart disease (ref. no)	-1.26	0.99	-0.07	0.205
Chronic painful condition (ref. no)	0.93	1.14	0.05	0.413
Glaucoma/cataract (ref. no)	-0.01	1.08	0.00	0.990
Medication knowledge	0.19	0.12	0.08	0.120
Number of chronic conditions	0.18	2.32	0.01	0.939

*Note*: ^*^p < 0.05, ^**^p < 0.01, ^***^p < 0.001

## Discussion

Understanding individuals’ experiences and burdens associated with medication use has increasingly been recognised as an integrated component of delivering patient-centred care [21]. This study examined the factors associated with medication burden among community-dwelling older people with multimorbidity. From a multidimensional perspective, our study provided insights into the role of various individual and psychosocial factors in affecting medication burden. Study findings have shown that older people with multimorbidity who had poor satisfaction with medication treatments, great disease burden, low medication self-efficacy, depression, and polypharmacy were more likely to have a great perceived burden of medication treatments.

Our sample had an overall low level of medication burden. The average score of the first four items of the TBQ was slightly lower than those reported in previous studies [12, 33, 46]. The multivariate analysis showed that nearly half of the variance of medication burden could be explained by the variables input in the model. The proportion of explained variance was higher compared with those (range: 16.0–38.9%) in previous studies, which only considered individuals’ demographic and clinical characteristics [12, 26, 47]. This difference could be accounted for the inclusion of psychosocial factors, which contributed to the additional explanation of medication burden.

Medication treatment satisfaction and medication burden are important aspects of patients’ subjective experiences of medication use. Medication treatment satisfaction involves patients’ evaluation of the process and the results of treatment experiences, whereas medication burden emphasises the efforts required of patients to live with and manage medications [48, 49]. Both aspects can impact one’s perspective and behaviour towards medications, which consequently influence patients’ decision-making process and health outcomes [50]. In our final model, the satisfaction with medication treatment exhibited the highest regression coefficient, demonstrating its considerable role in affecting medication burden. Previous studies have reported a bivariate association between medication treatment satisfaction and medication burden or treatment burden [19, 22, 51]. Based on a multivariate-adjusted model, our study further extended knowledge of the statistically significant association between them. An individual’s satisfaction and feelings during the process of taking medication may determine whether the patient perceives the hassles and problems caused by medication treatments as burdens and affect the extent of the impact this burden has on one’s adherence behaviour and health outcomes. An understanding of medication treatment satisfaction may provide important insights into the perceptions of older people experiencing being overburdened by their medication treatments.

The concept of self-efficacy has been the central focus within various social behavioural theories and is regarded as the most potent predictor of behaviour change and disease self-management [38]. Our study shows that higher medication self-efficacy was independently associated with lower medication burden. Medication self-efficacy reflects an individual’s beliefs and confidence in his or her ability to execute medication self-management activities [38]. Older people with high medication self-efficacy are more likely to overcome barriers and difficulties in medication use, such as coping with side effects, integrating medication treatments into daily life, and taking medication when travelling. A high level of medication self-efficacy can help patients better engage in medication treatments and perceive less burden associated with medication use. Previous studies have shown that self-efficacy in managing chronic diseases was a predictor of patients’ treatment burden [52, 53]. Our study further demonstrated their significant associations with respect to medication treatments. Healthcare professionals should help patients build medication self-efficacy to minimise the negative impact of medication burden on patients’ lives and health.

Medication-related knowledge, beliefs and social support were not found to be independently associated with medication burden. These findings, however, are not consistent with other studies. Evidence has shown that individuals who had limited health literacy, difficulties in understanding health information, or negative relationships with social network members were at great risk of high treatment burden [49, 53, 54]. Although these variables did not independently influence medication burden in our study, their impacts on medication self-efficacy should not be ignored. According to the Information-Motivation-Behavioural Skills model, individuals’ medication-related knowledge, personal motivation, and social motivation (i.e., one’s perception of social norms of medication taking and perceived social support) can directly influence self-efficacy in performing medication-related activities. Moreover, medication self-efficacy plays a role in mediating the effects of knowledge and motivation on one’s behaviour [30, 55]. Therefore, medication-related knowledge, beliefs, and social support may exert their effects on medication burden through their influence on medication self-efficacy. However, the potential indirect effects of these variables on medication burden have not been tested in previous research and may warrant verification in future studies.

Older people with a high level of disease burden are likely to struggle with the workloads imposed by multiple medical treatments. The presence of multimorbidity can also lead to poor physical and mental functions, further diminishing older people’s capacity to self-manage. It is not surprising that disease burden was significantly positively associated with medication burden in the multivariate analysis. Previous studies have shown that multiple chronic diseases, longer disease duration, greater disease severity, and poor self-rated health were significantly associated with higher perceived treatment burden [26, 27, 53, 56–58]. Moreover, our study found that only depression was significantly associated with medication burden, while all individual physical conditions were non-significant. Similarly, a previous study also identified that depression, dementia, or severe mental health problems were significantly associated with patients’ treatment burden, whereas no individual physical condition was found to be associated [58]. This finding may indicate the important role of mental problems in worsening the medication burden. However, some studies reported that having diabetes, atrial fibrillation, or hypertension significantly predicted higher medication burden or overall treatment burden [26, 29]. Therefore, whether an individual physical condition may contribute to high medication burden requires further investigation.

Our study did not identify any association between patients’ demographic factors and medication burden. Results from previous studies are inconsistent. Several studies showed that certain demographic factors, such as age, sex, employment conditions, and marital status, were associated with medication burden [13, 27]. However, a recent study reported that patients’ treatment burden did not differ across demographic factors [53]. The authors also proposed that demographic factors may act as a moderator to buffer the effect of patients’ self-management capacity on treatment burden. Therefore, the specific role of these factors needs further exploration.

### Limitations

This study has several limitations. Firstly, the cross-sectional data limited the ability to infer causal relationships. As medication burden is a dynamic process that evolves with the emergence of new treatments or the progression of chronic conditions [59], future studies should investigate the prospective associations between study variables and medication burden. Secondly, participants were recruited from two urban community health centres with high health insurance coverage. This may limit the study findings to populations who are living in rural areas with limited access to health services or those who are not insured. Thirdly, our study might not involve older people with high disease or treatment burden, who were less likely to have additional time and energy to participate in voluntary research. Fourthly, due to the secondary nature of the data used, we did not include interpersonal factors such as family issues and consultation styles, as well as institutional/infrastructural factors such as quality of healthcare services and continuity of care [10]. Further studies are required to include these factors for a more thorough understanding of medication burden.

## Conclusions

This study shows that higher medication self-efficacy and medication treatment satisfaction were independently associated with decreased medication burden in older people with multimorbidity, while depression, polypharmacy and greater disease burden were related to increased medication burden. Identifying psychosocial factors associated with medication burden may assist in detecting older people feeling overwhelmed by their medication treatments. Medication self-management support should be provided for older people with multimorbidity to enhance their capacity for performing medication treatments and relieve their medication burden. Healthcare professionals should exert more effort into understanding medication-related satisfaction and experiences from the patient perspective to deliver tailored psychosocial interventions for patients overwhelmed by medication burden. Psychological interventions might play a role in relieving depressive symptoms and therefore reducing medication burden.

### Electronic supplementary material

Below is the link to the electronic supplementary material.

Supplementary Material 1

## Data Availability

The data that support the findings of this study are available from the corresponding author but restrictions apply to the availability of these data, and they are not publicly available. Data are however available from the authors upon reasonable request and with permission of the corresponding author, upon reasonable request.

## Abbreviations

ANOVA: One-way analysis of variance
BMQ: Beliefs about medication questionnaire
SD: Standard deviations
TBQ: Treatment burden questionnaire
VIF: Variance inflation factor
