# Supplementary material for: Psychosocial factors associated with medication burden among community-dwelling older people with multimorbidity
Source: BMC Geriatr. 2023 Nov 14;23:741. doi: 10.1186/s12877-023-04444-6 (PMC10648314; doi:10.1186/s12877-023-04444-6)
Supplement: Supplementary file 1 — Supplementary Material 1 [file 12877_2023_4444_MOESM1_ESM.docx]

**Table S1**. Multiple linear regression including all potential factors associated with medication burden

| Variables | Unstandardised coefficients | | Standardised  coefficients  Beta | p |
| --- | --- | --- | --- | --- |
|  | B | Standard error |  |  |
| Global satisfaction with medication treatments | -0.21 | 0.04 | -0.34 | < 0.001^***^ |
| Disease burden | 0.92 | 0.36 | 0.25 | 0.012^**^ |
| Medication self-efficacy | -0.30 | 0.07 | -0.23 | < 0.001^***^ |
| Polypharmacy (ref. no) | 2.49 | 1.23 | 0.13 | 0.044^*^ |
| Depression (ref. no) | 1.23 | 0.51 | 0.14 | 0.017^*^ |
| Age | 0.03 | 0.07 | 0.02 | 0.708 |
| Female | -0.06 | 0.99 | 0.00 | 0.948 |
| Marriage status (ref. married) | 0.01 | 1.13 | 0.00 | 0.994 |
| Coronary heart disease (ref. no) | -1.15 | 1.02 | -0.06 | 0.261 |
| Chronic painful condition (ref. no) | 1.05 | 1.18 | 0.05 | 0.374 |
| Glaucoma/cataract (ref. no) | 0.26 | 1.18 | 0.01 | 0.828 |
| Medication knowledge | 0.14 | 0.12 | 0.06 | 0.242 |
| Medication beliefs |  |  |  |  |
| Necessity of medication | 0.11 | 0.11 | 0.05 | 0.330 |
| Concerns about medication | -0.01 | 0.10 | -0.01 | 0.918 |
| Harm of medication | -0.23 | 0.20 | -0.07 | 0.247 |
| Overuse of medication | 0.13 | 0.16 | 0.05 | 0.826 |
| Medication social support | 0.79 | 0.55 | 0.08 | 0.149 |
| Number of chronic conditions | 0.22 | 2.40 | 0.01 | 0.926 |

*Note.* ^*^p<0.05, ^**^p<0.01, ^***^p<0.001; A significant regression equation was found (F(18,235) = 11.462, p < 0.001), with an R^2^ of 0.467.
